# **Correction**: Hyperprolactinemia with antipsychotic drugs in children and adolescents

**DOI:** 10.1186/1687-9856-2013-13

**Published:** 2013-08-16

**Authors:** Arlan L Rosenbloom

**Affiliations:** 1 Department of Pediatrics, University of Florida College of Medicine, Children's Medical Services Center, 1701 SW 16th Avenue, Gainesville, FL 32608, USA

## Correction

Three errors have been noted by the author in an article published in this journal in 2010 [1], all three in paragraphs three and four under the heading:

“**4. Effects of psychotropic drugs on prolactin secretion”:**

1. Reference 19 in the original article [1] (Frazier JA, et al. Risperidone treatment for juvenile bipolar disorder: a retrospective chart review. *J Am Acad Child Adolesc Psychiatry.* 1999;38:960–5) was quoted as indicating that one of the 9 of 11 outpatients aged 4–17 years treated with risperidone who developed hyperprolactinemia had amenorrhea and one had gynecomastia. In fact, the only hyperprolactinemia associated complication reported was the one case of amenorrhea; there were no instances of gynecomastia.

2. Reference 23 in the original article [1] (Bunker MT, Marken PA, Schneiderhan ME, Ruehter VL. Attenuation of antipsychotic induced hyperprolactinemia with clozapine. *J Am Acad Child Adolesc Psychiatry* 1997; 7:65–9) was mistakenly described as reporting a single patient treated with risperidone who developed galactorrhea and had resolution of the problem when switched to clozapine. In fact, the subject had been treated with thioridazine (haloperidol).

3. The author described reference 24 of the original article [1] (Findling et al. Prolactin levels during long-term risperidone treatment in children and adolescents. *J Clin Psychiatry* 2003; 64:1362–9), as a retrospective study of prolactin levels and hyperprolactinemia attributable side effects from 5 clinical trials involving 592 children and adolescents aged 5–15 years that yielded a 2.2% frequency of side effects of gynecomastia, amenorrhea, or galactorrhea, quoting the published report. However, the frequency noted in the article was based on Findling et al. erroneously using the denominator for the entire 5-15-year-old population (n = 592) rather than the subpopulation that excluded boys over 10 years of age that they separately analyzed, in order to avoid confounding by adolescent gynecomastia (n = 360). It was within this smaller population that the 13 adverse events occurred. The correct frequency for hyperprolactinemia attributable side effects in these studies, therefore, should be 3.6% for this subpopulation and for the entire 5-15-year-old population in which 30 hyperprolactinemia attributable side effects occurred, 5%.

The first error, though regrettable, removes a single case that does not affect the conclusion that, “First-generation antipsychotics, particularly haloperidol, and the second-generation antipsychotic drugs, most prominently risperidone, appear to be associated with greatest risk for hyperprolactinemia: some treated individuals developing hyperprolactinemia will have galactorrhea, amenorrhea, or gynecomastia. [1]” The second error, in which haloperidol rather than risperidone was implicated, remains consistent with the conclusion. Correction of the more substantial and significant third error serves to reinforce this conclusion.
